# Tracking motion trajectories of individual nanoparticles using time-resolved current traces†
†Electronic supplementary information (ESI) available: Experimental details and adsorption energy. See DOI: 10.1039/c6sc04582k



**DOI:** 10.1039/c6sc04582k

**Published:** 2016-12-12

**Authors:** Wei Ma, Hui Ma, Jian-Fu Chen, Yue-Yi Peng, Zhe-Yao Yang, Hai-Feng Wang, Yi-Lun Ying, He Tian, Yi-Tao Long

**Affiliations:** a Key Laboratory for Advanced Materials , Institute of Fine Chemicals , East China University of Science and Technology , Shanghai , P. R. China . Email: tianhe@ecust.edu.cn ; Email: ytlong@ecust.edu.cn; b State Key Laboratory of Chemical Engineering Centre for Computational Chemistry , Research Institute of Industrial Catalysis , East China University of Science and Technology , Shanghai , P. R. China

## Abstract

Single nanoparticle (NP) electrochemical measurements are widely described, both theoretically and experimentally, as they enable visualization of the electrochemical signal of a single NP that is masked in ensemble measurements. However, investigating the behavior of individual NPs using electrochemical signals remains a significant challenge. Here we report experiments and simulations demonstrating that multiple distinct motion trajectories could be discerned from time-resolved current traces by dynamic Monte Carlo simulations. We show that continuous monitoring and quantification of electrochemical oxidation of individual AgNPs using a low-noise electrochemical measurement platform produce significantly distinguished current traces due to the size-dependent motions of AgNPs. Our findings offer a view of the electrochemical signals of individual NPs that are largely different from that in the literature, and underscore the significance of motion behaviors in single NP electrochemistry.

## Abstract 2

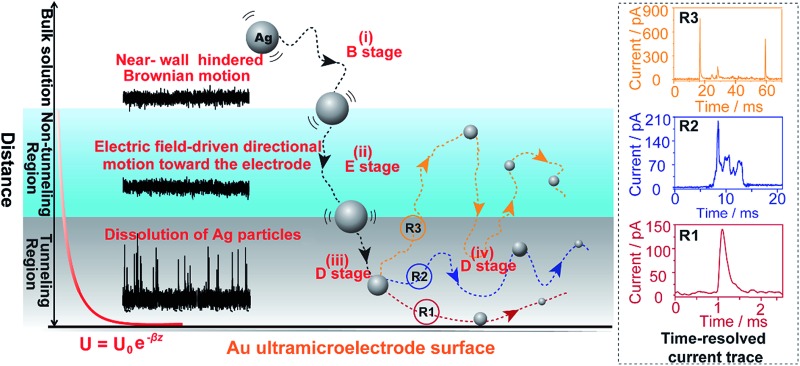
We report experiments and simulations demonstrating that multiple distinct motion trajectories of individual nanoparticles can be discerned from time-resolved current traces.

## Introduction

Single nanoparticle (NP) electrochemical measurements enable visualization of the electrochemical signal of a single NP that is masked in ensemble measurements, providing direct transduction of charge or electron transfer in dynamic single NP processes with extremely high sensitivity.^1–6^ However, the transient nature and ultralow amplitude of electrochemical signals from individual NPs represent a major challenge.^7–13^ A typical solution includes a small-sized ultramicroelectrode (UME) to reduce both background current and collision frequency^6–11,17^ and a highly-sensitive measurement system to resolve these signals.^14-16^ Despite the ongoing efforts to obtain the transient signal of an individual NP, low-resolution signals are often cited as a critical barrier to the progress of single NP electrochemical applications due to the difficulty of identifying well-defined signals among the noise.^18,19^

Over the past several decades, examples of single NP electrochemical measurements include electrocatalytic amplification^20–23^ and electrochemical oxidation^23–25^ of single metal oxide or metal NPs by detecting the current transients as individual NPs collide with a UME. Considerable experimental and theoretical effort has been devoted to studying the shape and statistical properties of these transients, which have significant practical applications for analyzing the size, structure, and catalytic characteristics of individual NPs.^20–28^ Investigations into the effects of NPs at conductive interfaces have yielded the information that NPs are either adsorbed onto or rebounded off the electrode surface.^29–34^ Recently, transport-reaction processes of individual nanoparticles were also investigated using electrochemistry coupled with *in situ* optical microscopy^19^ or scanning electrochemical cell microscopy^14^ during electrochemical impact. However, to date, a clear understanding of the motion behavior of individual NPs on the nanoscale still needs further exploration.

**Fig. 1:**
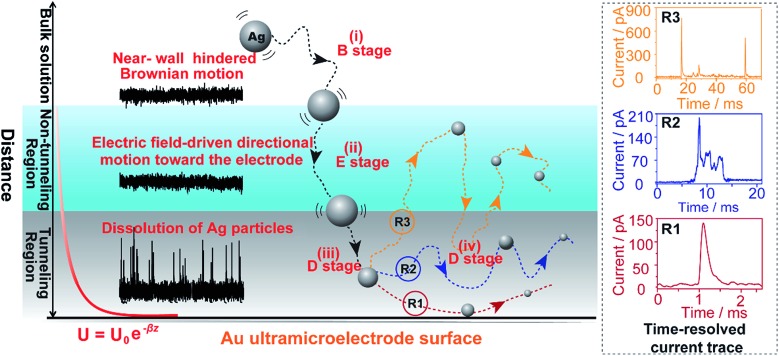
Schematic illustration of the motion trajectories of individual AgNPs using time-resolved current traces. The theoretical model divides the space above the near-wall region of the Au UME into three regions: the bulk solution, non-tunneling region, and tunneling region. Not to scale.

Here, we explore multiple distinct motion trajectories by investigating time-resolved current traces during the collision process of individual AgNPs onto an electrode surface using dynamic Monte Carlo simulations (Fig. 1). We successfully monitor and quantify the electrochemical oxidation of individual AgNPs using a low-noise electrochemical measurement platform, producing significantly resolved current traces. The high accuracy of the proposed current trace makes it possible to track the motion behavior of individual AgNPs as a function of the dwell time. A semi-quantitative theoretical model explicitly invoking adsorption energy and the different regions above the electrode was developed to account for the motion trajectories corresponding to the oxidation behavior of AgNPs using density functional theory (DFT) calculations. The common elementary routes involved in AgNP oxidation on Au UME surfaces include (i) hindered-diffusion in the near-wall region, (ii) electric field-driven directional motion towards the electrode in the non-tunneling region, (iii) directional motion and then adsorption accompanied by oxidation of Ag in the tunneling region, and (iv) adsorption or removal of the partially oxidized NP from the electrode due to the effects of stochastic diffusion, depending on the adsorption energy.

## Experimental

### Low-noise electrochemical measurement platform

A low-noise measurement platform that integrates a preamplifier with a current signal acquisition system^35^ was used to extract detailed information of electrochemical behavior at the single NP level. The amplifier's internal low-pass Bessel filter was set as 3 kHz. By introducing a high-performance preamplifier, we extended the signal bandwidth by at least one order of magnitude, providing more accurate evaluation of brief events. Compared with commercial devices, this integrated ultrasensitive current measurement platform has a current resolution as high as pico-ampere and an enhanced temporal resolution of sub-millisecond, which enabled us to observe precise electrochemical measurements of the relative timing of collision events. Data were acquired at a sampling rate of 250 kHz by using an A/D convertor and home-designed PC running software. Data analysis was performed using a second-order-differential-based calibration method to correct the region of the current signals and accurately measure the duration. This calibration method improves the accuracy of the local threshold approach for the determination of the starting and stopping point of current signals without requiring extra software. Meanwhile, the current amplitude was evaluated using an integration method that provided advantages in accurately recovering the heights of the signals. This self-developed integrated software system was an efficient tool for locating signals and determining their amplitudes and duration time, which could be performed automatically. The histograms of the current amplitudes, charges and durations were measured using this integration software.^36^ Second-order Gaussian functions were used to fit these histograms. To obtain a pico-ampere signal, all measurements were performed in a shielded room to minimize electromagnetic and radiofrequency interference.

### DFT calculations

All the spin-polarized calculations were performed using a Perdew–Burke–Ernzerhof functional within the generalized gradient approximation using the VASP code.^31–33^ The project-augmented wave method was used to represent the core–valence electron interaction.^39,40^ To model the Au (111) surface of a Au electrode, a four-layer *p*(3 × 3) slab corresponding to 36 Au atoms and a vacuum layer of 25 Å was applied. A 3 × 3 × 1 *k*-point mesh was used. Au 5d^10^6s^1^ and Ag 4d^10^5s^1^ electrons were treated as valence electrons. The valence electronic states were expanded in plane wave basis sets with an energy cutoff of 500 eV, and the force convergence criterion in the structure was set to 0.05 eV Å^–1^.

### Adsorption energy calculation method

The total adsorption energy (*E*totad) for Ag atoms was derived from the DFT calculation at 0 K, relative to the Ag particle, which is defined as*E*totad = *E*_Ag/Au_ – *E*_Au_ – *E*_Ag_where *E*_Ag/Au_, *E*_Au_, and *E*_Ag_ are the energies of the AgNP adsorbed on the Au electrode, the Au electrode and the AgNP. The *E*_ad_ of the Ag atoms with different coordination numbers was determined from *E*totad by averaging the surface-adsorbed Ag atoms. The more negative *E*_ad_ is, the more strongly the Ag atom binds to the Au surface. A negative adsorption energy signifies an exothermic reaction upon Ag adsorption. Considering the negligible contribution of water configurational entropy in theoretical energy calculations (Table S1†), we ignored the entropic effects in adsorption energy.

## Results and discussion

### Size-discriminated electrochemical oxidation of individual AgNPs

**Fig. 2:**
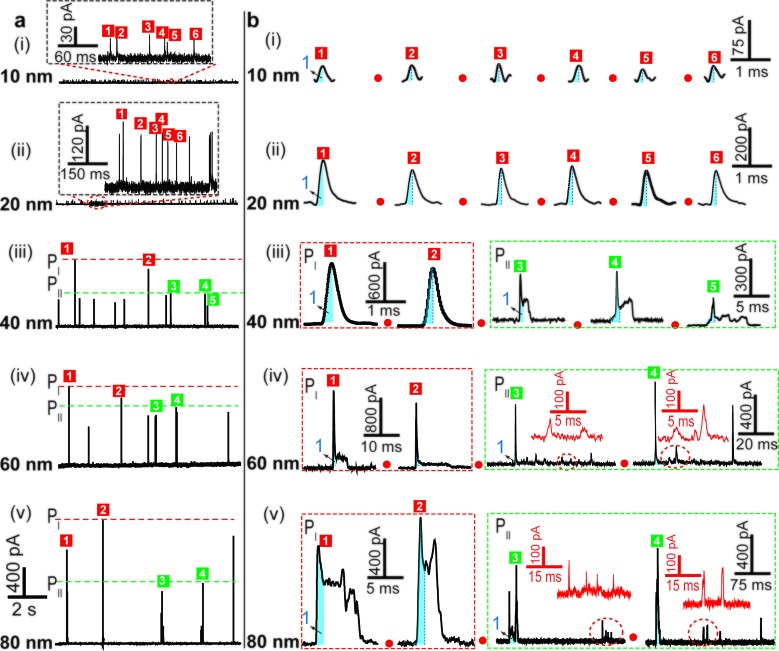
Time-resolved current traces of individual AgNPs with sizes of 10 nm (i), 20 nm (ii), 40 nm (iii), 60 nm (iv), and 80 nm (v). (a) Chronoamperometric profiles showing faradaic oxidation for individual AgNP collisions on the Au UME (diameter 12.5 μm) with the same scale bar. (b) Close-ups of the representative current traces (black line) and the corresponding magnified areas (red line) for the oxidation of individual AgNPs, with red dashed frames (P_I_) and green dashed frames (P_II_) added to represent the different current patterns. Blue-filled areas are defined as part 1 of the current traces. The scale bar is different for each size. Data acquired in 20 mM PB (pH = 7.4) at an applied potential of +0.6 V *vs.* Ag/AgCl wire in the presence of AgNPs.

**Fig. 3:**
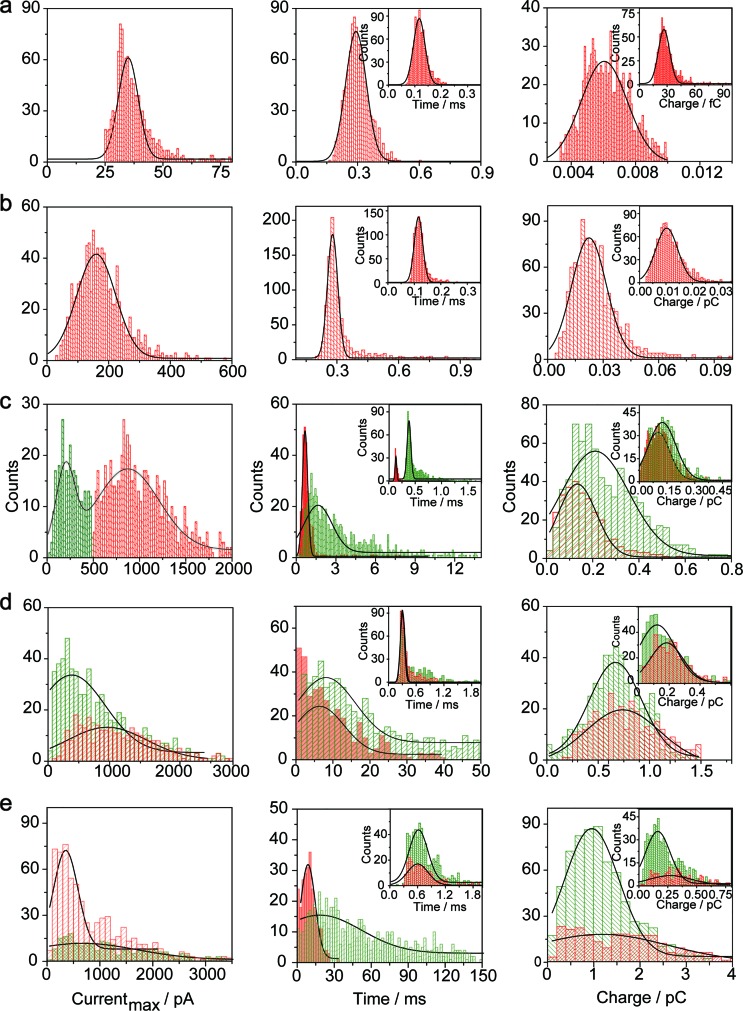
Histograms showing the distributions of the peak currents, durations and charges of AgNPs with a size of 10 nm (a), 20 nm (b), 40 nm (c), 60 nm (d), and 80 nm (e). The red bar and the green bar are denoted I and II, corresponding to the current patterns of P_I_ and P_II_, respectively (P_I_ and P_II_ are defined in the legend in Fig. 2). Inset: blue-filled part 1 of current traces. Black curves show Gaussian fits. The data were obtained from the chronoamperometry curves from a large population of oxidation events of individual AgNPs (more than 1000 events).

We examined the electrochemical oxidation behavior of individual AgNPs with different sizes at a given Au UME (diameter 12.5 μm) using a low-noise measurement platform. Transient currents were observed in the amperometric current–time curves when +600 mV *vs.* Ag/AgCl wire was held in 20 mM PB solution (pH = 7.4) containing AgNPs (Fig. 2a). Briefly, a faradaic oxidation reaction easily occurred when AgNPs randomly collided with the electrode, resulting in their dissolution. However, a bare Au UME was potentiostated, and no current signal was observed in a solution without AgNPs (ESI Fig. S2a†). An important feature of current transients is that the electrochemical oxidation varied with the size of the AgNPs. Additional evidence in support of this explanation is (1) no transient was observed when a lower electrode potential (*i.e.*, 0 mV, or +200 mV *vs.* Ag/AgCl wire) was applied (ESI Fig. S2b and c†); (2) as expected, the current amplitude of the transients increased with increasing particle size (Fig. 2a); (3) the collision frequency decreased significantly as the size increased (Fig. 2a). In all cases, the diameter of the Au UME is typically much larger than the AgNPs, and the particle concentration is selected in the pM range so that the probability of simultaneous events (*i.e.*, two AgNPs interacting with the Au UME at the same time) is very small. Nonetheless, there are some differences in optimum concentrations of different sized AgNPs for obtaining collisions at the single NP level (ESI Table S2†).

To further demonstrate that the transients obtained correspond to the oxidation of individual AgNPs, we integrated the quantity of faradaic charge transfer for each transient and estimated the particle size. Notably, the size distributions of 10 nm, 20 nm and 40 nm AgNPs obtained from the integrated charge and those obtained from dynamic light scattering measurements (ESI Fig. S4a–f†) exhibit good agreement, implying that our assumption was reasonable. However, the electrochemical measurement detected some large NPs (*i.e.*, 60 and 80 nm), which deviated from the size analysis (ESI Fig. S4g–j†). The apparent smaller diameter might be caused by the incomplete dissolution of AgNPs.^6,27^ In addition, experimentally observed frequencies are lower than the theoretically calculated ones by Fick's diffusion laws within a typical variation associated with stochastic measurements (ESI Table S2†), indicating that AgNP collisions on an Au UME are governed mainly by near-wall hindered-diffusion.^14,18^

### Time-resolved current traces of different sized individual AgNPs

The electrochemical oxidation process of an individual AgNP is expected to take a long time (>10 μs) for total dissolution. We reasoned that size-discriminated electrochemical behaviour would be transiently produced by low-noise electrochemical measurements. A characteristic single peak was observed within less than a millisecond for 10 nm AgNPs, exhibiting a symmetrical parabolic shape (Fig. 2b(i)). The distribution can be fitted well by a single Gaussian function (mean and standard deviation; Fig. 3), with a current amplitude of 35 ± 1 pA and a duration of 0.3 ± 0.1 ms. The small size of 10 nm AgNPs permits the probability of the timescale of a single encounter for total particle dissolution. We observed the duration of rising part 1 (blue-filled areas) and the corresponding charge accounted for approximately 50% of the complete oxidation (Table 1), demonstrating that the timescale of a single encounter is sufficient for a 10 nm AgNP to oxidize completely. In the case of 20 nm AgNPs, an asymmetrical current trace that initially rises quickly and decays at a slower rate^14,31^ was observed (Fig. 2b(ii)), yielding a current amplitude of 146 ± 2 pA and a longer duration of 0.5 ± 0.1 ms. We compared the duration and charge of rising part 1 with that of a single peak (Table 1), suggesting that the decay part makes a major contribution to the dissolution of 20 nm particles.

**Table 1:** Peak current (*I*_max_), duration (*T*) and charge (*Q*) of the electrochemical oxidation of individual AgNPs with sizes of 10 nm, 20 nm, 40 nm, 60 nm, and 80 nm^*a*^

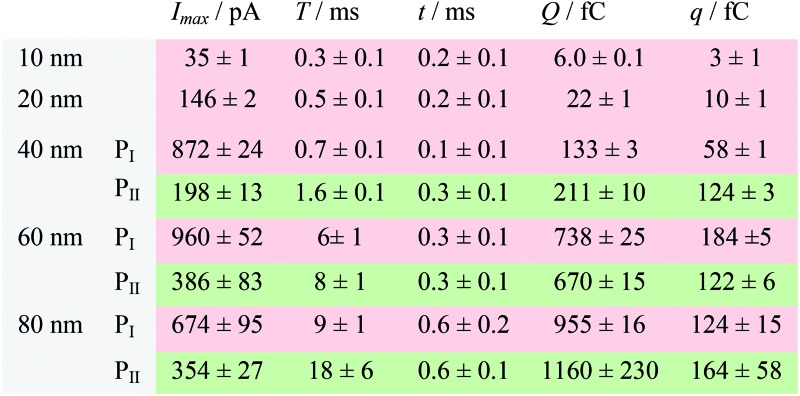

^*a*^Data were acquired in 20 mM PB (pH = 7.4) at an applied potential of +0.6 V *vs.* Ag/AgCl wire in the presence of AgNPs. Parameters of the current traces: peak current, *I*_max_; duration, *T*; charge, *Q*. Parameters of the blue-filled part 1 of the current traces: duration, *t*; charge, *q*. Errors are standard deviations of the means for *n* experiments. The values (means and standard deviations) can be fitted well by the Gaussian function. The red regions and green regions correspond to the current patterns of P_I_ and P_II_, respectively. The parameters are defined in the legend of Fig. 3.

Two current patterns always occur for the 40 nm AgNPs. As expected, a single large peak represents a single encounter but the amplitude was enlarged and the duration elongated (red frame, Fig. 2b(iii)), which is attributed to the charge associated with the number of Ag atoms in the particle. The results suggest that if the timescale of a single encounter (less than a millisecond) is adequate for the complete oxidation of a particular AgNP size, a single peak should be possible due to having similar oxidation behaviour. However, the predominant event observed for 40 nm AgNPs was a spike with an undulating terrain (green frame, Fig. 2b(iii)), showing a much smaller peak current (198 ± 13 pA) and a significantly increased duration of 1.6 ± 0.1 ms, thus leading to the approximate charge ranges for these two kinds of current pattern. In this tailing trace, the duration of the spike revealed that the timescale for a single encounter is on the order of magnitude of less than a millisecond, and the corresponding charge of blue part 1 is insufficient for a 40 nm AgNP to oxidize completely (Table 1).

However, current traces were observed almost exclusively as single peaks for the oxidation of the 60 nm AgNPs. Obviously, a similar current trace appeared with a spike with an undulating terrain (red frame, Fig. 2b(iv)), in which there were obvious enhancements in the amplitude of 960 ± 52 pA and the duration of 6 ± 1 ms. Interestingly, a spike with a closely spaced cluster (green frame, Fig. 2b(iv)) was dominant for 60 nm, in which the amplitude of the first spike dropped to 386 ± 83 pA, while the duration increased to 8 ± 1 ms. In particular, the entire duration could be more than an order of magnitude longer than that of the first spike, which is attributed to the complete oxidation of the 60 nm particle *via* a series of stages, during each of which the AgNP is partially oxidized. For the 80 nm AgNPs, we first observed a series of large current blockades (red frame, Fig. 2b(v)) with an amplitude of 674 ± 95 pA and a duration of 9 ± 1 ms. The current blockade is similar to a spike with an undulating terrain, but the undulating terrain is dominant. Notably, more than 80% of the cases displayed a cluster of current spikes for the 80 nm AgNP (green frame, Fig. 2b(v)), corresponding to the consecutive encounters of individual AgNPs on the electrode. These species displayed a distinct level with the current amplitude at 354 ± 27 pA and a much longer duration of 18 ± 6 ms. In the 60 and 80 nm cases, the contribution from the first spike to the total dissolution of the particle decreased gradually with increasing size, which is attributed to multiple-encounters for the oxidation of larger particles. The duration of the first spike is also consistent with the timescale of a single encounter, but the amplitude of maximum current was independent. In addition, a relatively wide distribution of durations and a further increase of random encounter processes for namely 60 and 80 nm AgNPs could be attributed to the stochastic behaviour of large-sized AgNPs.

### Motion trajectories of individual NPs using time-resolved current traces

In the case of current transients, we would expect an exponential current decay with duration if AgNPs start to oxidize only when the particles directly come into contact with the Au UME surface.^31^ However, the current traces obtained for individual AgNPs began to flow and increasingly reach a peak and decay with different fluctuations. Having obtained the above results, we are in the position to establish a semi-quantitative dynamic Monte Carlo simulation to estimate the corresponding motion trajectories of AgNPs from the current traces.

**Fig. 4:**
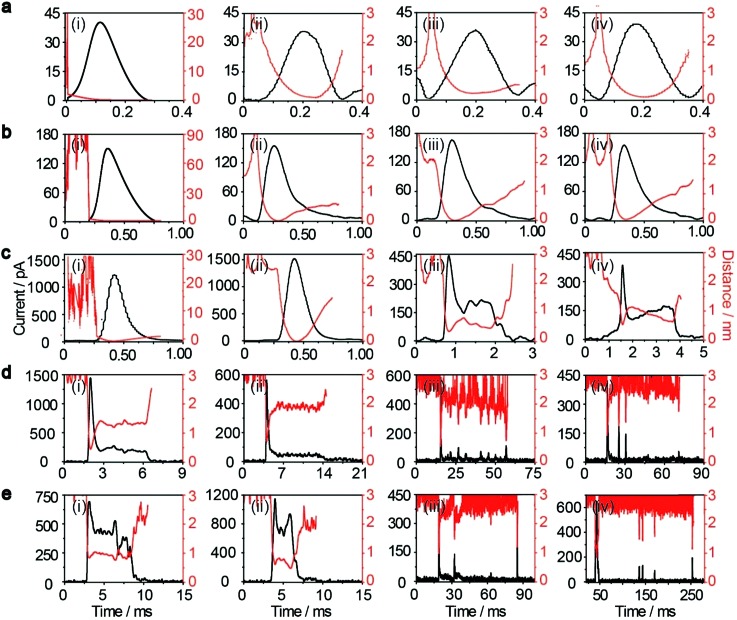
Motion trajectories (red line) of individual NPs and time-resolved current traces (black line) during electrochemical oxidation with AgNPs approaching the electrode surface simulated well using a dynamic Monte Carlo simulation. Current–time (left black ordinate) and distance–time (right red ordinate) of AgNPs with sizes of 10 nm (a), 20 nm (b), 40 nm (c), 60 nm (d), and 80 nm (e). The initial positions of the AgNPs are arbitrary.

In this theoretical model, we divide the space above the electrode into a tunneling region, a non-tunneling region, and the bulk solution (Fig. 1). AgNPs are subjected to random walks with solvent molecules in bulk solution, resulting in Brownian motion.^37,38^ As the particles approach sub-micron distances from the electrode surface, the diffusing AgNPs are affected by near-wall hindered-diffusion due to the boundary effect, reducing the velocity and increasing the time spent near the electrode.^18,34^ We resolved this behaviour by obtaining the displacement of an arbitrarily chosen AgNP as 
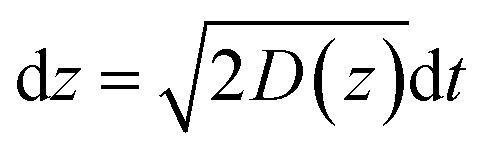
, where *D*(*z*) is the distance-dependent hindered-diffusion coefficient of the AgNPs 
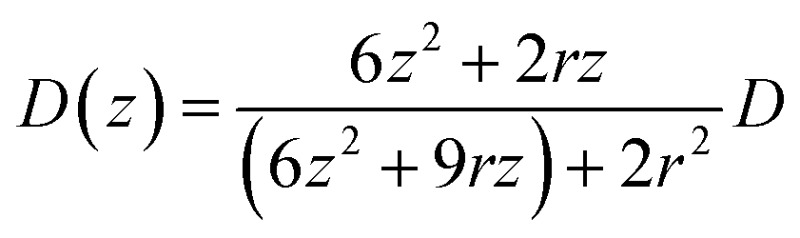
 with the diffusion coefficient *D* = *k*_B_ *T*/6π*ηr*, where *k*_B_ is the Boltzmann constant, *T* is the temperature, *η* is the dynamic viscosity of the solution and *r* is the AgNP radius. It is important to note that the hindered-diffusion is based on the consideration of the millisecond timescale of the individual AgNPs. Throughout this period, the AgNPs execute complex trajectories of stochastic motions that have a significant effect on particles close to the electrode. From simulation analysis, we observed that the particles stochastically oscillate *via* a rolling movement in the direction of the electrode (Fig. 4a–c(i)).

As the distance of a particle from the electrode approaches the nanometre scale, that is, the impact region, the directional movement of the particle is determined mainly by the electric potential with exponential dependence on the distance (*U* = *U*_0_e^–*z*/*λ*^, where *U*_0_ is the surface potential and *λ* is the Debye length) (Fig. S5†) and is affected by significantly hindered-diffusion which is simplified when simulated by stochastic motion with the frequency proportional to desorption rate (*k*_de_) and the particle size (*r*) (Fig. 1). Once the AgNP has come close to the impact region there is a high probability of several encounters before it moves out into the bulk solution due to the electric field, ensuring the complete oxidation of large particles.^28^ Theoretical prediction of the distance from the imaginary boundary of the impact region to the electrode surface indicates a length in the same range as the Debye length (∼3 nm). Here, we simply consider that the AgNP could undergo adsorption once the particle approached the electrode to within closer than a certain distance (the “scale of adsorption” ∼ 0.5 nm), while adsorbed particles wander due to the effects of stochastic diffusion, giving rise to a tumble motion. In analogy to adsorption and desorption processes, we consider the possibility of stochastic motion *p*_col_ = (*βr*)*k*_de_d*t* with desorption rate *k*_de_ = (*k*_B_ *T*/*h*)e^*E*_ad_/*k*_B_*T*^ according to the Arrhenius equation, where *h* is the Planck constant, and *E*_ad_ is the adsorption energy.^39^ Based on the DFT calculations, we estimated that *E*_ad_ is associated with AgNP size (*r*), as described by *E*_ad_ = –0.11/*r* – 0.64 within the generalized gradient approximation (ESI Fig. S6–S8†). Considering the continuously refreshed particle surface during the electrochemical oxidation of AgNPs, we did not take the effect of the surface capping agent (citrate) into consideration for *E*_ad_ in this DFT calculation. It is worthy of note that we developed the long range Ag–Au adsorption in terms of a through-space interaction and supposed the increase in *E*_ad_ with AgNPs approaching the electrode surface, resulting in the suppression of the stochastic motion of the particle.

When an AgNP is located within the tunneling subregion of the impact region, the AgNP is momentarily involved in the oxidation of Ag and enables a faradaic reaction (Fig. 3b). According to the first Faraday law, the faradaic current contributing to particle dissolution at any time *i*(*t*) during the electrochemical oxidation of an AgNP is given by
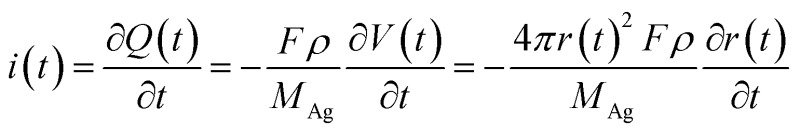
where *F* is Faraday's constant, *ρ* is the density of Ag, and *M*_Ag_ is the atomic molecular mass of Ag. The variation of rate with AgNP size is described by a zero-order rate law^12^ with exponential dependence on the tunneling distance (*z*) according to a tunneling constant,^31^ *β* ^–1^. It follows ∂*r*/∂*t* = –*v*_*r*_e^–*βz*^, where *v*_*r*_ is the electrochemical reaction rate. The tunneling distance can be calculated by *z* = *d* – *r* where *d* = *d*_0_ – *v*_*z*_ *t* is the displacement of AgNPs, given for the velocity of the *z*-component with time *v*_*z*_ = *v*_*z*0_ – *a*e^–*βz*^ *t* where *v*_*z*0_ is the initial velocity of the *z*-component entering the impact region. Distinct motion patterns are expected for different sized AgNPs owing to their size-dependent adsorption. In other words, the adsorption energy of a Ag atom on the Au UME surfaces may be a good descriptor for the motion trajectories of a particle. Qualitatively, if the adsorption energy of Ag is too large, the particle desorption would be suppressed, localizing the partially oxidized particle near the surface of the Au UME (R1 in Fig. 1). For the small sizes (10 nm and 20 nm), AgNPs adsorb temporarily and then rebound off the electrode due to the large adsorption energy so that the stochastic motion of the particle was suppressed, thus, the particles have simple trajectories (Fig. 4a and b). The desorbed particles still mediate oxidative dissolution and contribute to the current measured at the Au UME. Similar motion behaviour followed by a single encounter was observed for a small portion of the 40 nm particles (Fig. 4c(i and ii)). However, despite the large current amplitude, the duration is still shorter than a millisecond, indicating that the millisecond timescale is sufficient for particle motion from the imaginary boundary of the tunneling region to the electrode surface under the electric field-driven stochastic motions.

Weak adsorption of the large AgNPs is advantageous for particle desorption. That is, *k*_de_ is sufficiently large enough to overcome adsorption and induce the desorption of adsorbed particles from Au (111), leading to tumbling motion near the electrodes. Theoretically, two modes of tumbling motion can be classified: (i) the particle is always confined in the tunneling region (R2 in Fig. 1), and (ii) the particle moves away to the non-tunneling region and comes back again to the tunnelling region/electrode surface due to the attraction of the electric field (R3 in Fig. 1). Two kinds of processes are related to the particle size, depending on the size-controlled displacement of the stochastic motions. For a given current pattern, a spike with an undulating terrain, in which the current traces keep fluctuating in an undulating terrain but do not return to the baseline until total dissolution occurs, indicates that the AgNP begins to move towards the electrode and recede, then fluctuates in the tunneling region (Fig. 4c(iii and iv), d and e(i and ii)). Adsorption no doubt still exists during the fluctuation process; however, while an increase in desorption will accentuate the stochastic motion, it will at the same time increase the timescale of dissolution by reducing the reaction rate of the AgNPs. The widths of the undulating terrains seem uncertain, also suggesting the stochastic nature of the tumbling motion.

A representative trajectory for a series of consecutive millisecond spikes was observed, corresponding to sequential multistep “collision” events occurring on the Au UME, namely, the partially reacted NPs recede to the non-tunneling region and return again to the electrode surface several times during the full experimental timescale, besides some maintain fluctuation in the tunneling region during their entire oxidation. This behaviour is dependent on the increased likelihood of further collisions with the electrode once a dynamically moving AgNP has already approached closely. The simulation results also indicate that a particle has a longer timescale due to the tumbling motion from the bottom to the top in the impact region, especially for more adhesion/desorption events compared to confined fluctuations in the tunneling region (Fig. 4d and e(iii and iv)).

To exclude the possibility that the current responses observed were possibly affected by precipitation/dissolution of Ag_3_PO_4_ in a phosphate buffer solution,^41,42^ we also performed the electrochemical oxidation of 60 nm AgNPs in NaNO_3_ solution. As shown in Fig. S9,† two current patterns are still observed for the dissolution of 60 nm AgNPs in NaNO_3_ solution. Similar to the 60 nm AgNPs in 20 mM phosphate buffer (pH = 7.4), the current patterns in NaNO_3_ solution almost exclusively exhibit a spike with and undulating terrain. This demonstrates that the oxidation process in our system and current traces could be attributed to multiple distinct motion trajectories during the process of individual AgNP collisions on the electrode.

To validate the hypothesis that adsorption and desorption events did occur during the electrochemical oxidation of the AgNPs, we further scrutinized the detailed current traces as a function of temperature. The variation tendency of the number ratio of event II to event I (ratio = *N*_II_/*N*_I_) decays with temperature, implying that the motion behaviour of individual AgNPs became more stochastic as the temperature increased (ESI Fig. S10†). The temperature-controlled current patterns agree with *k*_de_ following the Arrhenius equation in our theoretical model, providing a thermodynamic basis for placing the *E*_ad_ as a fundamental property of the particle motion trajectories. The current amplitudes and durations from the theoretical simulations compare well with the measured results, also demonstrating that our theoretical model is reliable.

## Conclusions

Here, we explore the utility of dynamic Monte Carlo simulations by calculating, to our knowledge for the first time, multiple distinct motion trajectories, which could be discerned from time-resolved current traces during the process of individual AgNP collisions on an electrode. We show that continuous monitoring and quantification of the electrochemical oxidation of individual AgNPs using a low-noise electrochemical measurement platform, producing significantly distinguished current traces due to the size-dependent motion behaviors of the AgNPs. Our results demonstrate the possibility that through-space electron transfer occurs over the timescale from sub-millisecond to tens of milliseconds. In the next step, we will consider the bandwidth limitation of the existing commercial potentiostats and exploit a self-designed, high-bandwidth electrochemical instrument for optimizing single entity electrochemical measurements.
