# Supplementary material for: Tracking motion trajectories of individual nanoparticles using time-resolved current traces
Source: Chem Sci. 2016 Dec 12;8(3):1854–61. doi: 10.1039/c6sc04582k (PMC5424808; doi:10.1039/c6sc04582k)
Supplement: Supplementary file 1 [file SC-008-C6SC04582K-s001.pdf]

Electronic Supplementary Material (ESI) for Chemical Science.
This journal is © The Royal Society of Chemistry 2017

Supporting Information

Motion trajectories of individual nanoparticles using time-resolved current traces

Wei Ma,[a‡] Hui Ma,[a‡] Jian-Fu Chen,[b‡] Yue-Yi Peng,[a] Zhe-Yao Yang,[a] Hai-Feng Wang,[b] Yi-lun Ying,[a] He Tian[a]* and Yi-Tao Long[a]*

[a]Key Laboratory for Advanced Materials & Institute of Fine Chemicals, East China University of Science and Technology, Shanghai, P. R. China.

[b]State Key Laboratory of Chemical Engineering Centre for Computational Chemistry & Research Institute of Industrial Catalysis, East China University of Science and Technology, Shanghai, P. R. China.

State Key Laboratory of Chemical Engineering

Centre for Computational Chemistry and

Research Institute of Industrial Catalysis

East China University of Science and Technology

130 Meilong Road, Shanghai, 200237 (P.R. China)
State Key Laboratory of Chemical Engineering
Centre for Computational Chemistry and
Research Institute of Industrial Catalysis
East China University of Science and Technology
130 Meilong Road, Shanghai, 200237 (P.R. China)
‡These authors contributed equally.

Table of Contents

1. Materials --2
Materials--2
Instrumentation--2
2. Methods --3

Modified Au Electrode with AgNPs---3
Size of the collided AgNP from the integrated charge--------------------------3
Theoretical collision frequencies and diffusion coefficients of AgNPs-------3

2. Tables ---4
Table S1---4
Table S2---4
Table S3 --4

3. Figures --5
Figure S1---5
Figure S2---5
Figure S3 ---6
Figure S4--7
Figure S5--8
Figure S6 ---9
Figure S7--10
Figure S8--11
Figure S9--12
Figure S10---13

Materials

Materials

Silver nanoparticles (AgNPs), average nominal diameters of 10, 20, 40, 60, and 80 nm, were obtained by nanoComposix, Inc. (San Diego, CA, USA). All analytical grade reagents were purchased from Sigma-Aldrich. All electrodes for electrochemical experiments were purchased from Shanghai Chenhua Co., Ltd., China. N_2 (99.998%, prepurified) was obtained from Cryogenic Gases (Detroit, MI). Millipore water (Milli-Q system) purified to a resistivity of 18.2 MΩ·cm with a UHQ II system (Elga) was used to prepare all the solutions. Phosphate buffer solution (PB) was prepared using Na_2HPO_4 and NaH_2PO_4. The electrolyte solutions were prepared with Millipore water and deaerated by purging with N_2 atmosphere for 5 min before experiment.

Instrumentation

For Differential-pulse voltammetry measurements, an Au electrode (diameter 2 mm) was used as the working electrode, Ag/AgCl as the reference electrode while a Pt wire electrode acted as a counter electrode (at 25 °C). Chronoamperometric curves were performed using low-noise current measurement platform with the two electrode cell system. The electrochemical cell was consisted of an Au UME (12.5 μm) and an Ag/AgCl reference electrode. The solution of electrochemical cell contained only 20 mM PB buffer without any other electrolyte. All electrochemical measurements were performed in a double Faraday cage at 25 °C. The pH value of a solution was measured by a PHS-3C (Switzerland Mettler Toledo Delta 320 pH meter). Light scattering analysis was performed with a Zetasizer Nano-ZS

Instrument (ZEM4228) from Malvern Instruments using the protocols provided by the supplier.

Methods

1. Modified Au Electrode with AgNPs

Drop casting was used to AgNPs modify an Au electrode (diameter 2 mm) to observe the stripping voltammetry of different sized AgNPs. This method was involved with the process of casting a drop of AgNPs suspension on the surface of Au electrode followed by evaporation of the solvent. In the experiment of anodic stripping voltammetry, 2 μl of freshly prepared AgNPs suspension of 1μM was dropped on the surface of an Au electrode, and then the electrode was left in N_2 atmosphere for about 20 min to evaporate the solvent. Differential-pulse voltammetry was subsequently used for electrochemical measurements with the modified Au electrode in 20 mM PB buffer as supporting electrolytes.

2. Size of the collided AgNPs from the integrated charge

To further demonstrate the obtained current transients correspond to the oxidation of individual AgNPs, we integrated the quantity (Q) of Faradic charge transfer in each transient. From the integrated charge, we can estimate the size of the collided AgNP. In this work, we assume that all NPs are spherical (independent of their sizes), and that single AgNP is completely oxidized during the collision process. The NP radius (r_{NP}) can be calculated through the equation 1 below[1]:

$$r_{NP} = \sqrt[3]{\frac{3QAr}{4\pi nF\rho}} \qquad (1)$$

where Q is the integrated charge from current transient, n is the number of electrons transferred per Ag atom, Ar is the atomic molecular mass of Ag of 107.9 g mol^{-1}, F is Faraday's constant, and ρ is the density

of Ag of 10.5×10^6 g m^{-3}. Cumulative all-point diameter histograms of AgNPs for a large population of oxidation events (more than 1000 events) based on equation 1 is presented in Figure S3.

3. Theoretical collision frequencies and diffusion coefficients of AgNPs

The theoretical collision frequency of AgNPs at the Au UME can be estimated based on equations for a purely diffusive NP flux, based on Fick's diffusion laws. The collision frequency (j_{NP})[2] governed by the AgNP diffusion can be calculated by equation 2,

$$j_{NP} = 4D_{NP}C_{NP}N_A r_{elec} \quad (2)$$

Where C_{NP} is the concentration of AgNPs, N_A is Avogadro's constant (6.02×10^{23} mol^{-1}), and r_{elec} is the diameter of the Au UME. The diffusion coefficient of an AgNP (D_{NP}) can be determined from the Stokes-Einstein equation, equation 3,

$$D_{NP} = \frac{k_B T}{6\pi\eta r_{NP}} \quad (3)$$

where k_B is the Boltzmann constant (1.381×10^{-23} $J{\cdot}K^{-1}$), T is temperature (298 K), η is the dynamic viscosity of water (8.94×10^{-4} Pa·s at 298 K), and r_{NP} is the radius of an AgNP.

Tables

Table R1. Calculated DFT energies of E_{Au}, E_{Ag}, $E_{Ag/Au}$ and E_{ad} at different coordination number n_c with (sol) and without (gas) the solvent effects in combination with the polarizable continuum model.

	n_c	E_{Au}	E_{Ag}	$E_{Ag/Au}$	E_{ad}^{tot}	n_{ad}	E_{ad}
Gas	**9**	-110.45	-92.20	-208.64	-5.98	9	-0.66
Sol	**9**	-110.43	-92.20	-208.74	-6.11	9	-0.68
Gas	**6**	-110.45	-81.75	-198.11	-5.91	6	-0.98
Sol	**6**	-110.43	-81.76	-198.20	-6.01	6	-1.00
Gas	**3**	-110.45	-69.15	-181.02	-1.42	1	-1.42
Sol	**3**	-110.43	-69.19	-181.13	-1.51	1	-1.51

We evaluated DFT energies of E_{Au}, E_{Ag}, $E_{Ag/Au}$ and E_{ad} with (sol) and without (gas) the contribution of water configurational entropy from the DFT calculation. As shown in Table S1, the changes in calculated value of DFT energies are negligible, indicating entropic contribution plays only a slight role in theoretical energy calculation. Thus, we ignored the entropic effects in our DFT calculation.

Table S2. Charge (*Q*) and collision frequencies (J_{NP}) of AgNP collision based on the theoretically calculated values and experimental observation[a] at the optimization concentration (*C*).

	C / pM	*Q* / pC	J_{NP} / NP s^{-1}	
**10 nm**	99[b]	0.006 ± 0.001[c]	18 ± 3.2[c]	72.8[d]
**20 nm**	84[b]	0.022 ± 0.001[c]	12 ± 2.1[c]	30.9[d]
**40 nm**	26.7[b]	0.174 ± 0.006[c]	1.1 ± 0.3[c]	4.91[d]
**60 nm**	12.5[b]	0.642 ± 0.013[c]	0.8 ± 0.3[c]	1.53[d]
**80 nm**	4.49[b]	0.954 ± 0.036[c]	0.5 ± 0.2[c]	0.46[d]

[a]Data obtained from the chronoamperometry curves for a large population of electrochemical oxidation events of individual AgNPs (more than 1000 events); [b]Concentrations of AgNPs obtained the optimum possibility of collision at the single NP level; [c] Experimental results. Errors are standard deviations of the means for *n* experiments; [d]Theoretically calculated values. Date acquired in 20 mM PB (pH = 7.4) at an applied potential of +0.6 V *vs* Ag/AgCl in the presence of AgNPs.

Table S3. Populations of coordination numbers and atoms for AgNP faces, edges and vertexs in icosahedral structure.

**Type**	**Face**	**Edge**	**Vertex**
**Number of coordination numbers**	9	8	6
**Number of types**	20	30	12
**Number of atoms per type**	(n-1)(n-2)/2	n-1	1
**Total atoms per type**	10(n-1)(n-2)	30(n-1)	12
**Total coordination numbers per type**	90(n-1)(n-2)	240(n-1)	72

Figures

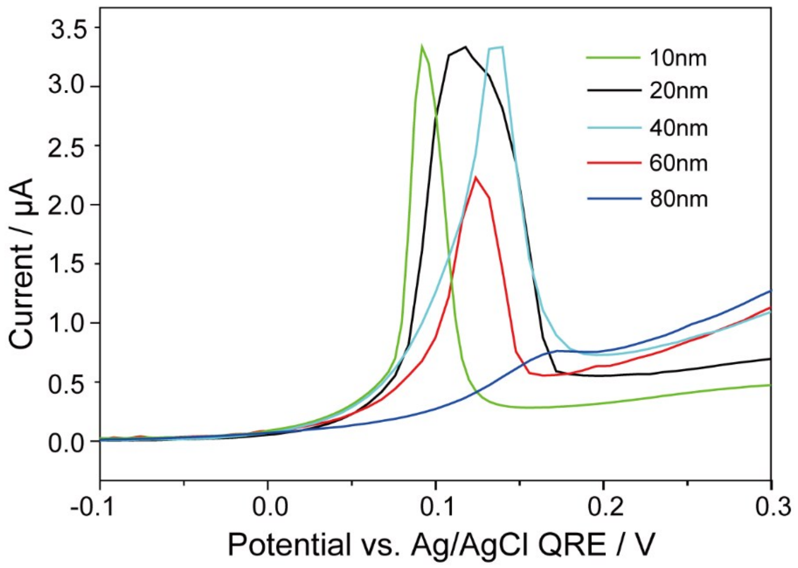

Figure S1. Differential-pulse voltammetry obtained in 20 mM PB (pH = 7.4) at a scan rate of 50 mV s^{-1} on a Au electrode (diameter 2 mm) coated with AgNPs of 10, 20, 40, 60 and 80 nm.

Anodic stripping voltammetry was performed with five samples of commercially available AgNPs with various sizes of 10, 20, 40, 60 and 80 nm. Differential pulse voltammetry (DPV) measurements were carried out using a AgNP-modified Au electrode (diameter 2 mm) in 20 mM PB buffer to observe the stripping voltammogram of AgNPs (**Fig. S1**). Each experiment was repeated three times to ensure reproducibility. **Fig.** S1 shows DPVs for different sized AgNPs with corresponding t peak potentials (E_p) of 278, 320, 400, 358 and 491 mV, respectively. The negative anodic current corresponds to the reaction of Ag^0 - 1e$^-$ $\rightarrow$ Ag^+.

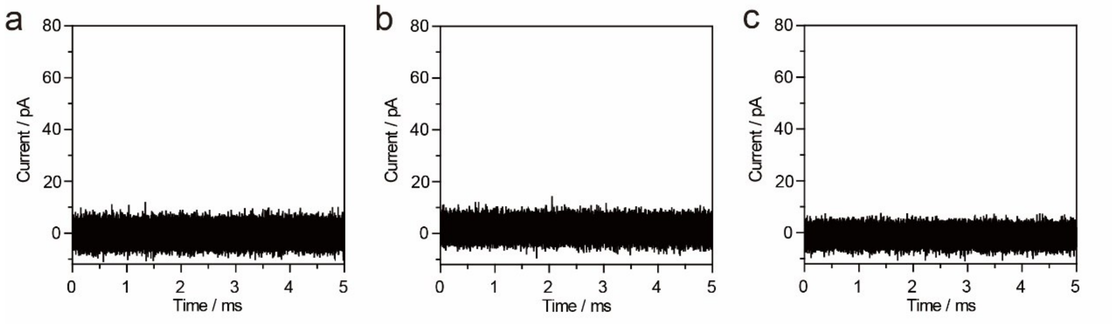

Figure S2. A five-second chronoamperometric profile on the Au UME (diameter 12.5 μm) in 20 mM PB (pH = 7.4) containing (a) no AgNPs at +600 mV *vs* Ag/AgCl and (b) 20 nm AgNPs at applied lower potentials of 0 mV *vs* Ag/AgCl and (c) +200 mV *vs* Ag/AgCl.

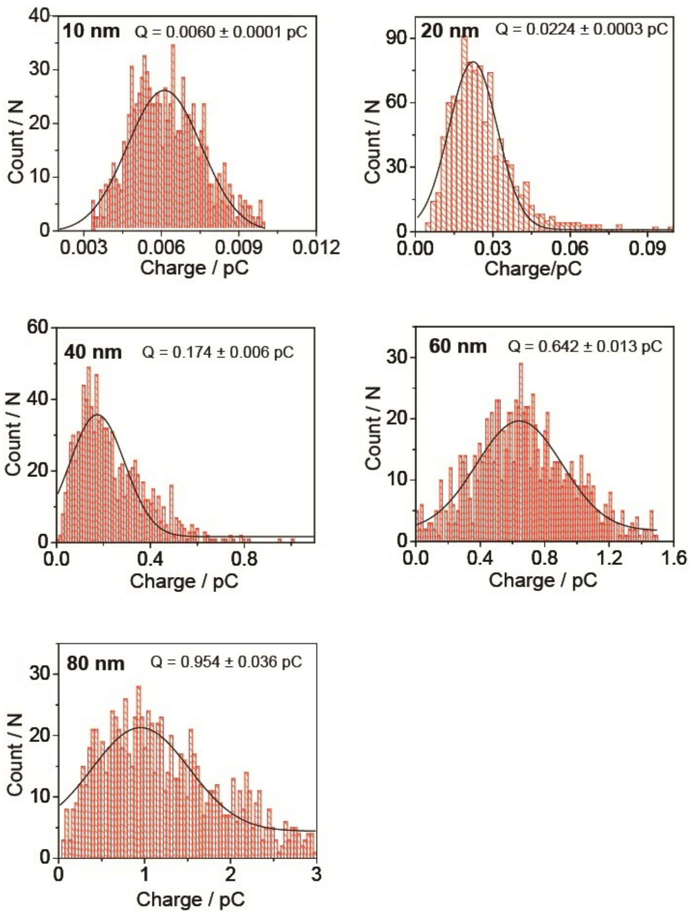

Figure S3. Histograms showing the charge distributions of different AgNPs sizes obtained from the chronoamperogrames.

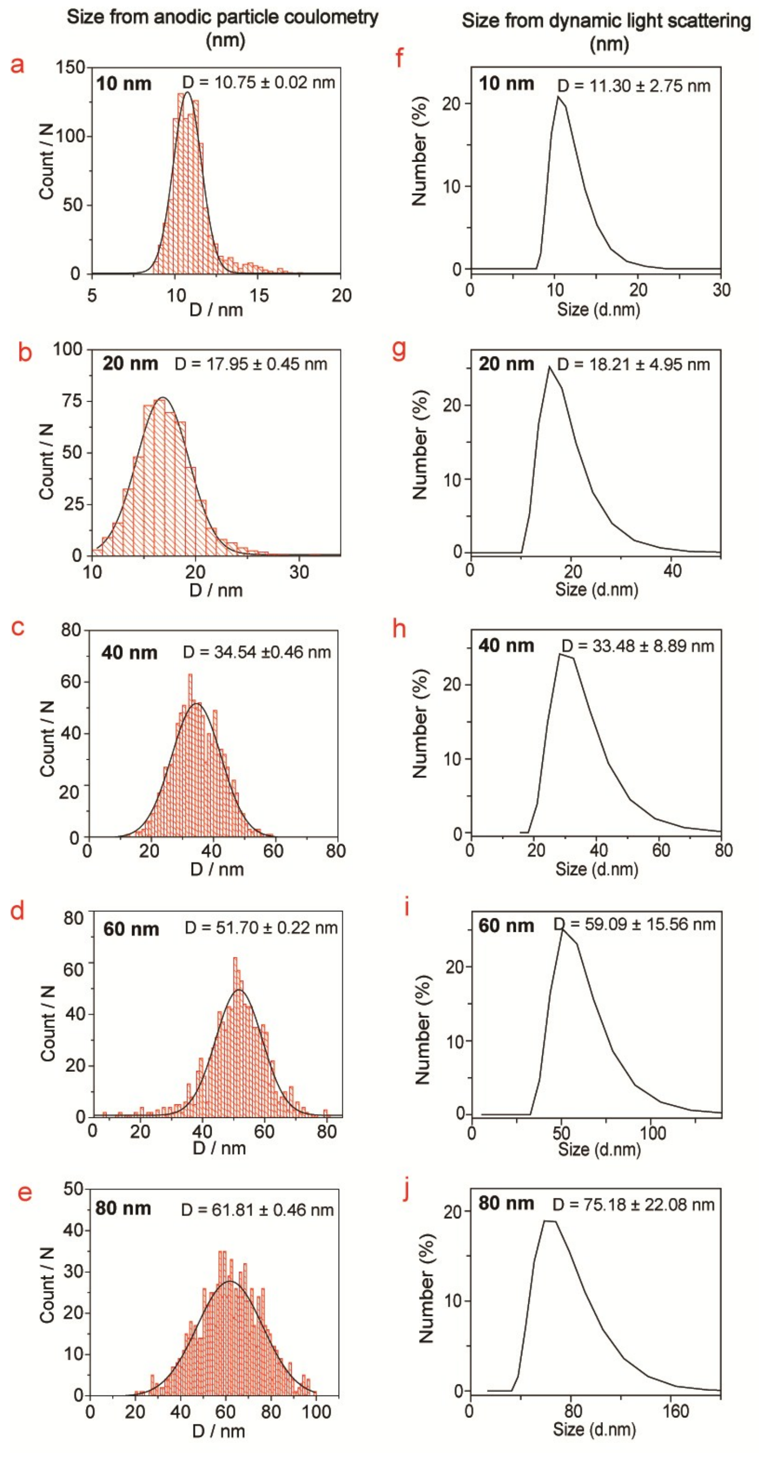

Figure S4. Size distributions of AgNPs with different sizes of 10 nm (a, f), 20 nm (b, g), 40 nm (c, h), 60 nm (e, i), and 80 nm (f, j). Left: Histograms showing the size distribution of AgNPs from the integrated charge for a large population of electrochemical oxidation events (more than 1000 events). Black curves show Gaussian fits. Right: Dynamic light scattering measurements of the hydrodynamic diameters of AgNPs with different sizes.

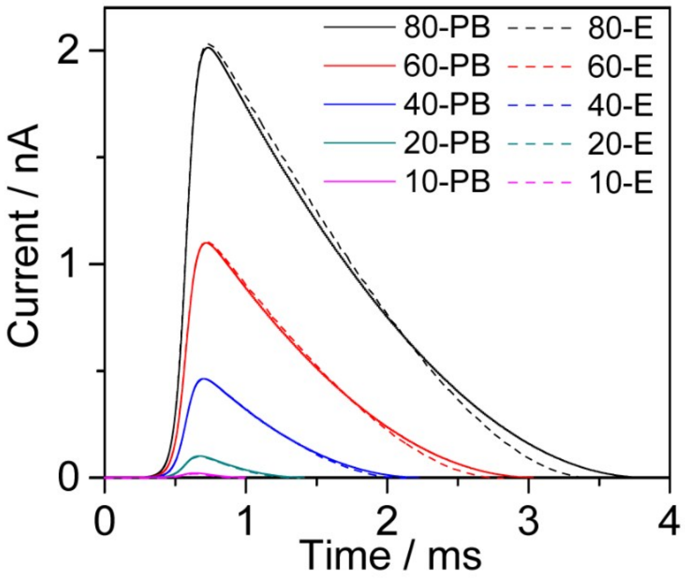

Fig. S5 Simulated current–time of AgNPs with sizes of 10 nm, 20 nm, 40 nm, 60 nm, and 80 nm using dynamic simulation by the electric potential model with the Poisson-Boltzmann equation (solid line) and the Exponential decay (dot line) under no stochastic behavior of AgNPs.

In order to evaluate a suitable potential-distance model, we investigated the simulated current–time of AgNPs with sizes of 10 nm, 20 nm, 40 nm, 60 nm, and 80 nm using dynamic simulation with a simple exponential decay ($U = U_0e^{-z/\lambda}$) and Poisson-Boltzmann equation ($U = 4k_BT/e\tanh^{-1}[\tanh[eU_0/4k_BT]e^{-z/\lambda}]$), where U_0 is the surface potential and λ is the Debye length. The dynamic simulation model also deliberately abandoned any stochastic behavior of AgNPs to obtain a better comparison of using different electric potential descriptions. From the simulated results of the amperometric current-time curves, a single trajectory, exhibiting an asymmetric and exponentially decaying transient, was observed for the complete electrochemical oxidation of a single AgNP (Fig. S5). Moreover, the current-time curve using an exponential decay model shows a similar behavior to that obtained with the Poisson-Boltzmann equation (Fig. S5). The potential approximation with an exponential decay model is therefore evidently sufficient to account for our experimental observations, especially for a semi-quantitative model.

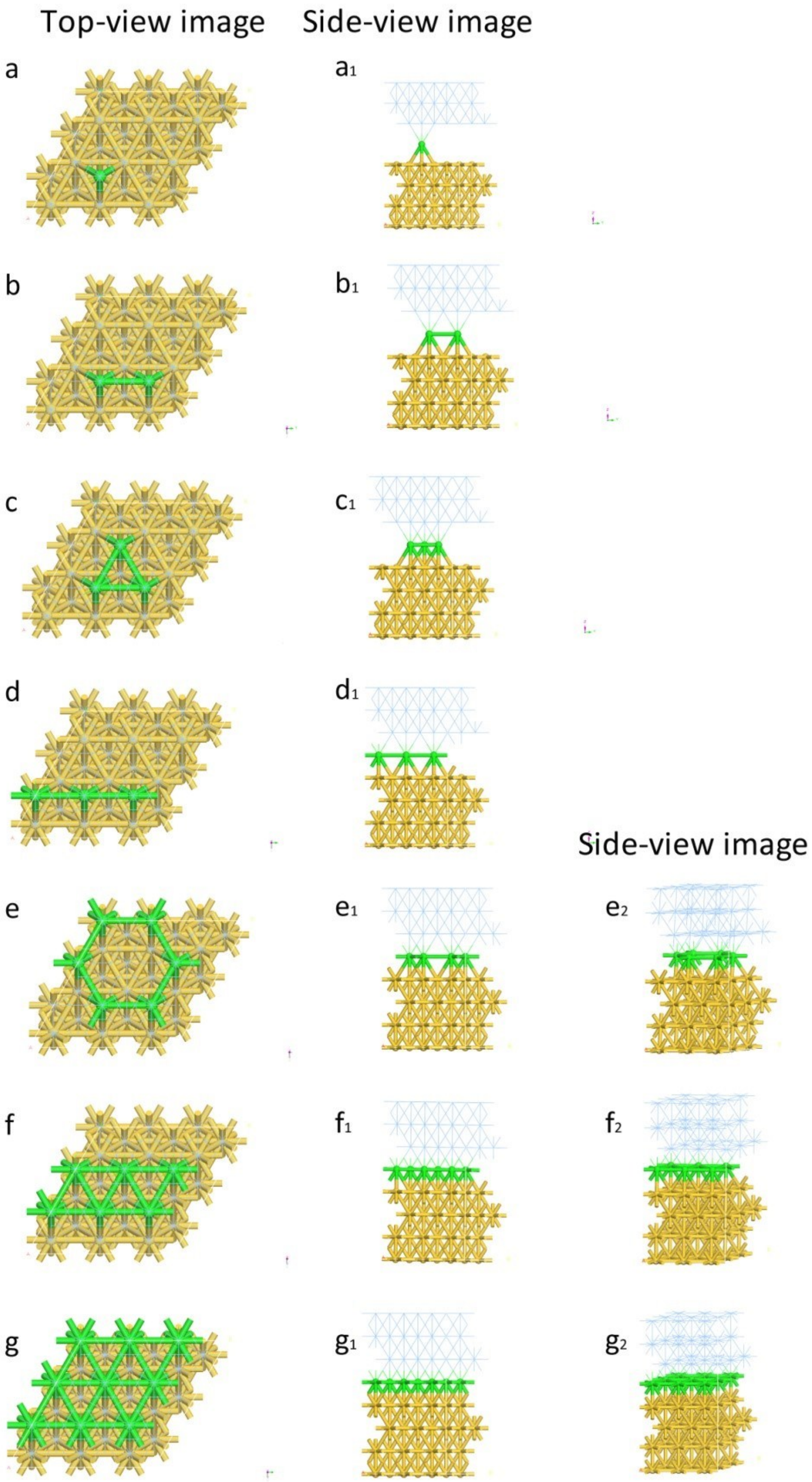

Figure S6. Adsorption configurations of Ag atoms with different coordination on Au (111) surface for DFT calculation.

We applied a first-principle calculation in combination with Ag atom absorption to the Au(111) surface, which provides an accurate description of the adsorption energy. We then analysed the origin of the bonding trends with particle size. A series of possible adsorption sites on the Ag atom of Au surface were checked. The energetically most stable adsorption configuration is shown in **Fig. S6** (denoted as Ag/Au), from which it can be seen that each Ag atom is uprightly adsorbed on the Au(111) surface with a different number of coordinated Ag atoms. Table S1 shows the calculated $E_{\mathrm{Ag/Au}}$, E_{Ag}, E_{Au} and E_{ad} with the coordination number (n_c).

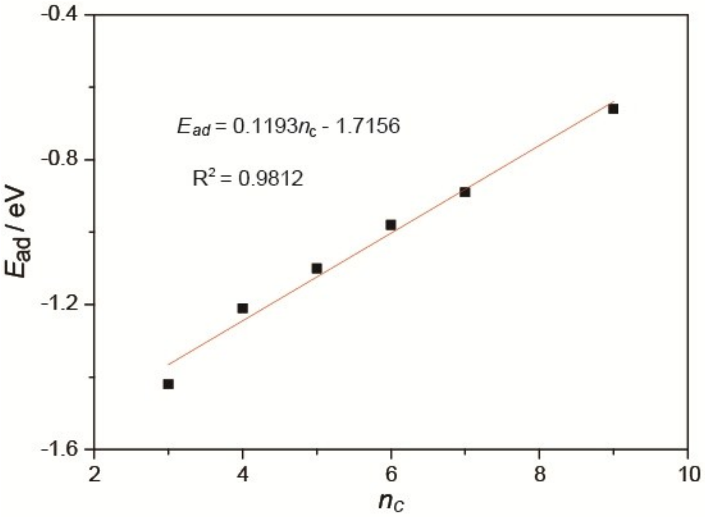

Figure S7. Calibration curve of the adsorption energy (E_{ad}) of each Ag atom *vs* the coordination number (n_c) absorbing on Au (111) surfaces. The line has been fitted by regression analysis, and the correlation coefficient is 0.9812.

Based on linear fit as regression analysis, we observed that the adsorption energy increased as the coordination number increased, exhibiting a good dependence from 3 to 9 (**Fig. S7**). In this study, the adsorption energies, E_{ad}, are based on the coordination numbers of the Ag atom (n_c), and are determined using $E_{\mathrm{ad}} = a{*}n_c + b = 0.1193{*}n_c - 1.7156$, where the correlation coefficient is 0.9812.

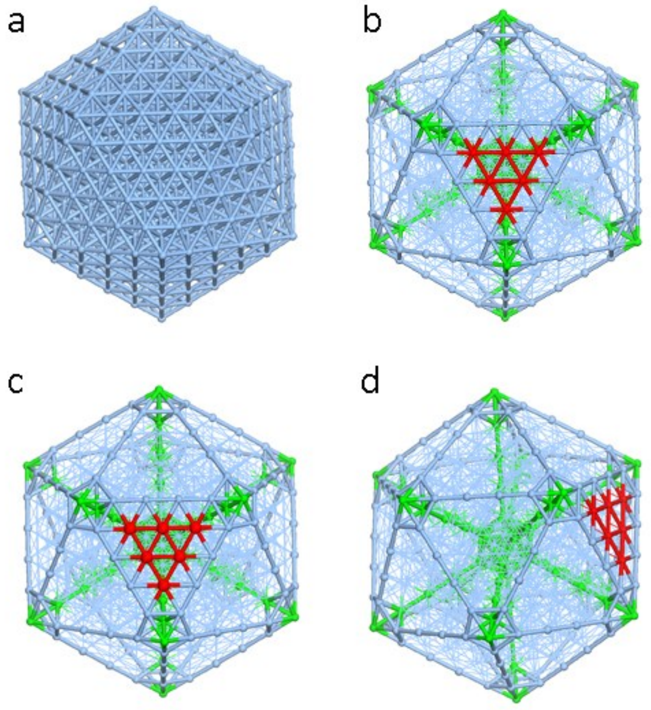

Figure S8. Icosahedral structure of AgNPs in a different orientation.

For the description of the theoretical basis, we approximated the AgNPs used in this work as icosahedral entities[6]. We further estimated the average coordination number (n_c) *vs* particle radius (r) using regular icosahedrons. We investigated the lowest-energy structures and the electronic properties of the icosahedrons using a generalized gradient approximation. The icosahedral structures of AgNPs in different orientations calculated with DFT are shown in **Fig. S8**. The icosahedral AgNPs are bounded by 20 orderly arranged triangular faces, and all of them are {111} planes of the face centre cubic structure. The populations of coordination numbers and atoms are given as functions of particle faces, edges and vertexs in icosahedral structure (**Table S3**). To determine the number of Ag layers in the regular icosahedrons, we defined $n = r/d_{Ag}$, where d_{Ag} is the distance between two Ag atoms and r is the AgNP radius. The calculation of the mean coordination numbers from the formulae in **Table S3** gives the following expressions for n_c, in terms of coordination number per atoms:

Total number of coordinations = 90 (n-1)(n-2) + 240(n-1) + 72 = 90n^2 -30n + 12

Total number of atoms = 10(n-1) (n-2) + 30(n-1) + 12 = 10n^2 + 2

Average number of coordinations (n_c) = (90n^2 -30n + 12)/(10n^2 + 2) = 9 – 3/n = 9 – 3d_{Ag}/r

Then, we can estimate the relationship between the adsorption energy (E_{ad}) and radius(r) by E_{ad} = a*(9 - 3d_{Ag}/r) + b = -0.11/r – 0.64. According to this equation, the adsorption energy (E_{ad}) decreased as the particle size increases, indicating that the adsorption is stronger for smaller AgNPs during collisions on the surfaces of electrode. All these effects are due to an increase in the number of Ag sites with low coordination numbers on the small particles. Similar trends also had been reported in Pt and Au nanoparticles by Nørskov's recent work[7], demonstrating our adsorption relationship is reliable.

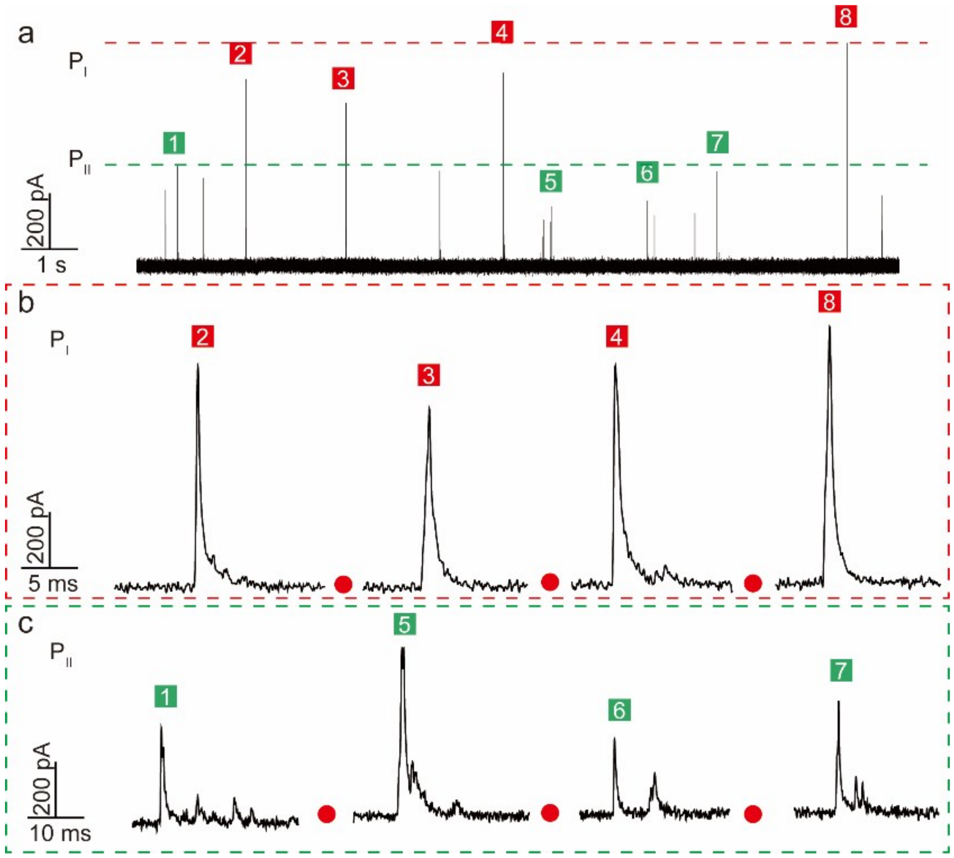

Figure S9. Time-resolved current traces of individual 60 nm AgNPs. a, Chronoamperometric profiles showing Faradaic oxidation for individual AgNPs collisions on the Au UME (diameter 12.5 μm) with the same scale bar. **b, c** Close-ups of the representative current traces for the oxidation of individual AgNPs, with red dashed frames (P_I) and green dashed frames (P_{II}) added to represent the different current patterns. Date acquired in 30 mM $NaNO_3$ at an applied potential of +0.6 V vs Ag/AgCl in the presence of AgNPs.

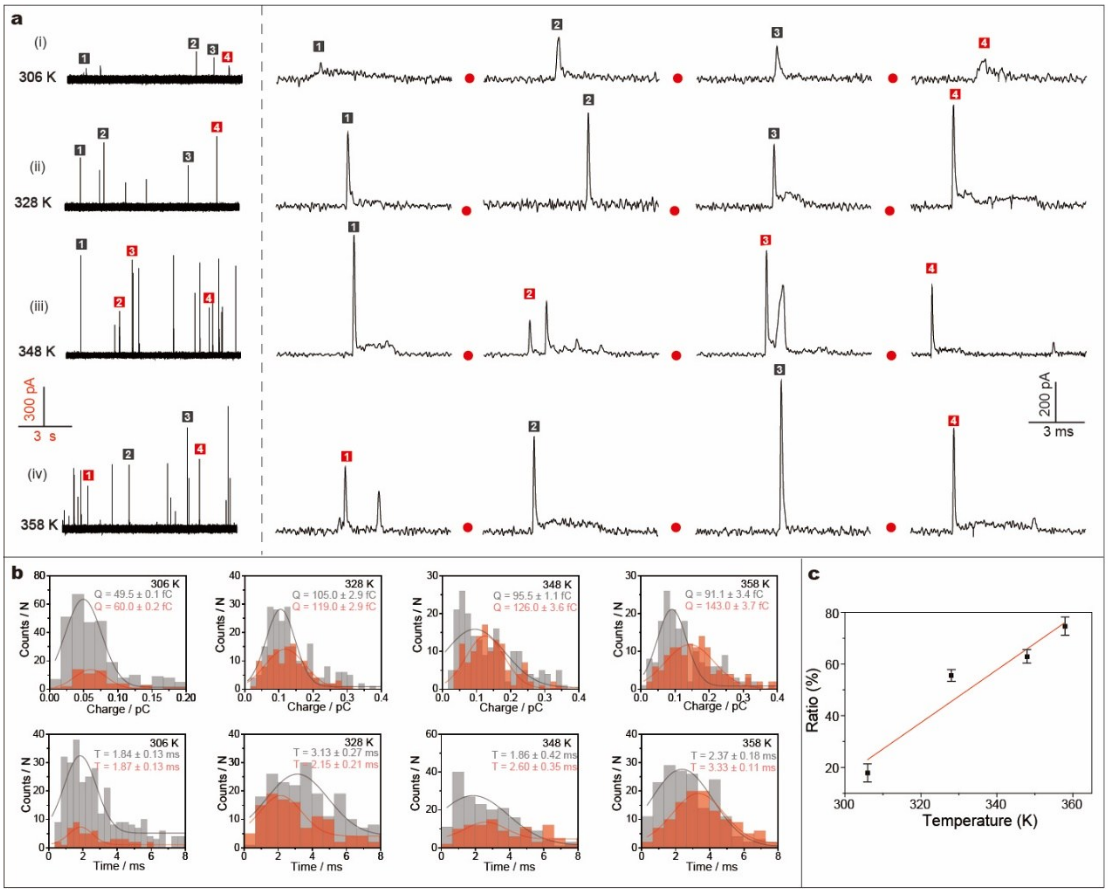

Figure S10. Temperature-dependent current patterns of individual AgNPs. **a**, Time-resolved current traces of individual AgNPs (40 nm) as a function of temperature at 306 K (i), 328 K (ii), 348 K (iii), and 358 K (iv). Left: chronoamperometric profiles showing Faradaic oxidation for individual AgNP collisions on the Au UME. Right: close-ups of representative current traces. Current traces including single peak and a spike with undulating terrain are defined event I (grey marks), while the current trace of a spike with a closely spaced cluster is defined as event II (red marks). **b**, Histograms showing the distribution of duration and charge for the oxidation of 40 nm AgNPs at different temperatures. The grey and red bars correspond to the different current patterns from event I and event II, respectively. **c**, The variation tendency of the number ratio of event II to event I (Ratio = N_{II}/N_{I}) *vs* temperature. The data were acquired in 20 mM PB (pH = 7.4) at an applied potential of +0.6 V *vs* Ag/AgCl in the presence of AgNPs.

A significant increase in the frequency of the current signals was observed as the temperature increased (**Fig. S10a**, left), further confirming the diffusion controlled collision process of AgNPs on the Au UME. Current patterns of AgNPs depend on the temperature of the surrounding medium. The significant decrease in charge at 306 K was ascribed to the deactivated electrode when electrolysis was conducted at low temperature[5]. However, narrow charge ranges were found at 328 K, 348 K and 358 K, i.e., from 0.1 pC to 0.15 pC, suggesting that 40 nm AgNPs were almost completely oxidised. Importantly, the duration significantly increased from 328 K to 358 K rather than at room temperature, demonstrating that more complex trajectories of AgNPs occurred during the electrochemical oxidation process (**Fig. S10b, c**).

Reference

1. H. S. Toh, K. Jurkschat, and Compton, R. G. The Influence of the capping agent on the oxidation of silver NPs: nano - impacts versus stripping voltammetry. *Chem. Eur. J.* 2015, **21**, 2998-3004.

2. A. J. Bard and L. R. Faulkner, Electrochemical Methods, Fundamentals and Applications; 2 ed.; John Wiley & Sons: New York, 2001.

3. B. K. Kim, J. Kim and Bard, A. J. Electrochemistry of a Single Attoliter Emulsion Droplet in Collisions. *J. Am. Chem. Soc.* 2015, **137**, 2343-2349.

4. O. S. Ivanova and Zamborini, F. P. Size-dependent electrochemical oxidation of silver nanoparticles. *J. Am. Chem. Soc.* 2010, **132**, 70-72.

5. N. B. Tahar and Savall, A. Electropolymerization of phenol on a vitreous carbon electrode in alkaline aqueous solution at different temperatures. *Electrochimica Acta* 2009, **55**, 465-469.
6. Q. Zhang, J. Xie, J. Yang and Lee, J. Y. Monodisperse icosahedral Ag, Au, and Pd NPs: size control strategy and superlattice formation. *Acs Nano* 2008, **3**(1), 139-148.

7. L. Li, F. Abild-Pedersen, J. Greeley and J. K. Nørskov, Surface tension effects on the reactivity of metal nanoparticles. *J. Phys. Chem. Lett.* 2015, **6** (19), 3797–3801.
